# Effects of the FIT Game on Physical Activity in Sixth Graders: A Pilot Reversal Design Intervention Study

**DOI:** 10.2196/13051

**Published:** 2019-06-18

**Authors:** Damon Joyner, Heidi Wengreen, Sheryl Aguilar, Gregory Madden

**Affiliations:** 1 Weber State University Ogden, UT United States; 2 Utah State University Logan, UT United States

**Keywords:** children, accelerometer, step count

## Abstract

**Background:**

The FIT Game is a low-cost intervention that increases fruit and vegetable consumption in elementary school children. For this study, the FIT Game was adapted into an intervention designed to increase children’s physical activity at school.

**Objective:**

We aimed to evaluate if the FIT Game could increase children’s physical activity relative to their baseline levels.

**Methods:**

A total of 29 participants were recruited from a sixth-grade classroom. An ABAB reversal design was used. Participants wore an accelerometer while at school during pre/postintervention baseline (A) and intervention (B) phases. During the FIT Game intervention, daily physical activity goals encouraged the class to increase their median daily step count above the 60th percentile of the previous 10 days. When daily goals were met, game-based accomplishments were realized.

**Results:**

Children met their activity goals 80% of the time during the intervention phases. Physical activity at school increased from a median of 3331 steps per day during the baseline to 4102 steps during the FIT Game phases (*P*<.001, Friedman test).

**Conclusions:**

Preliminary evidence showed that playing the FIT Game could positively influence children’s physical activity at school.

## Introduction

The prevalence of obesity has increased in recent years, and this condition affects more than one-third of children in the United States [1]. Childhood obesity has negative health consequences both during childhood [2-4] and, if not ameliorated, throughout the lifespan [5,6]. Meeting the recommended guidelines for daily physical activity (PA) reduces the risk for overweight, obesity, and chronic disease [7,8]. Increased activity also improves cardiorespiratory fitness, flexibility, and muscular strength [9]. These benefits extend into adulthood because childhood PA levels are predictive of adult PA levels [10-12]. Despite these short- and long-term benefits, PA declines from ages of 6-19 years [13], and many children in the United States do not meet the recommended 60 minutes of daily moderate to vigorous PA [8,14,15].

Addressing this public health crisis should include school-based interventions because most children in the United States spend 6-8 hours a day, 5 days a week, in school. As such, effective school-based interventions have the potential to help children develop healthy PA patterns that become habitual (ie, consistent daily initiation of PA without having to consciously remember to do so [16]). Within the public-school setting, interventions most likely to be widely adopted are those that positively influence healthy behavior while incurring minimal material and labor costs.

With these goals and constraints in mind, our research group developed the FIT Game, a low-cost game-based intervention designed to increase fruit and vegetable intake in elementary-aged children [17-19]. This intervention uses equipment already present in elementary schools in the United States (computer and projector) and employs video game design principles to motivate healthy eating. Because the FIT Game has consistently produced significant increases in healthy eating among children at school [17-19], we aimed to adapt it to the goal of increasing PA at school. In this single-case experiment, the FIT Game was played over 20 days with PA goals instead of healthy-eating goals. The experiment was conducted as a pilot study preliminary to future studies of more rigorous design.

## Methods

### Recruitment

All students (n=29) enrolled in the selected sixth-grade classroom were invited to participate. The classroom was selected because it was convenient and located in an elementary school in the Cache County, UT, school district. All the children in the selected classroom, and their parents/guardians, provided written consent to participate. The students were healthy and without physical disabilities that would constrain mobility. Of the students, 90% were Caucasian, 59% were female, and 26% of the children attending the school qualified for free or reduced lunch. All procedures were reviewed and approved by the Institutional Review Board for the Protection of Human Subjects at Utah State University.

### Instruments

Each child was assigned a unique wrist accelerometer (Fitbit Flex, San Francisco, CA). Students were instructed to wear the accelerometer on their nondominant wrist. The Fitbit Flex provides reliable PA data by reporting daily step counts [20]. As per the standard Fitbit function, tapping the accelerometer face produced illuminated dots, each representing 2000 steps, to a maximum of five dots, although children were not informed of this function or the meaning of the dots prior to the intervention. A projector available in the classroom was used to show FIT Game episodes on a screen in the front of the room. Each episode was accessed through Google Slides, cycled through the slides automatically, and lasted approximately 2 minutes. A personal computer equipped with Fitbit Connect (Fitbit Flex) was used to sync children’s accelerometers with their online Fitbit accounts.

### Intervention

The intervention was based on the FIT Game, which has been previously used to increase fruit and vegetable consumption of elementary school children [17-19]. The FIT Game is a science-fiction narrative in which the Field Intensive Trainees (the FITs) are tasked with finding and capturing three villainous members of the Vegetation Annihilation Team (VAT) before they can cause planetary destruction. The object of the game for the sixth-grade players was to help the FITs complete this task.

**Figure 1:**
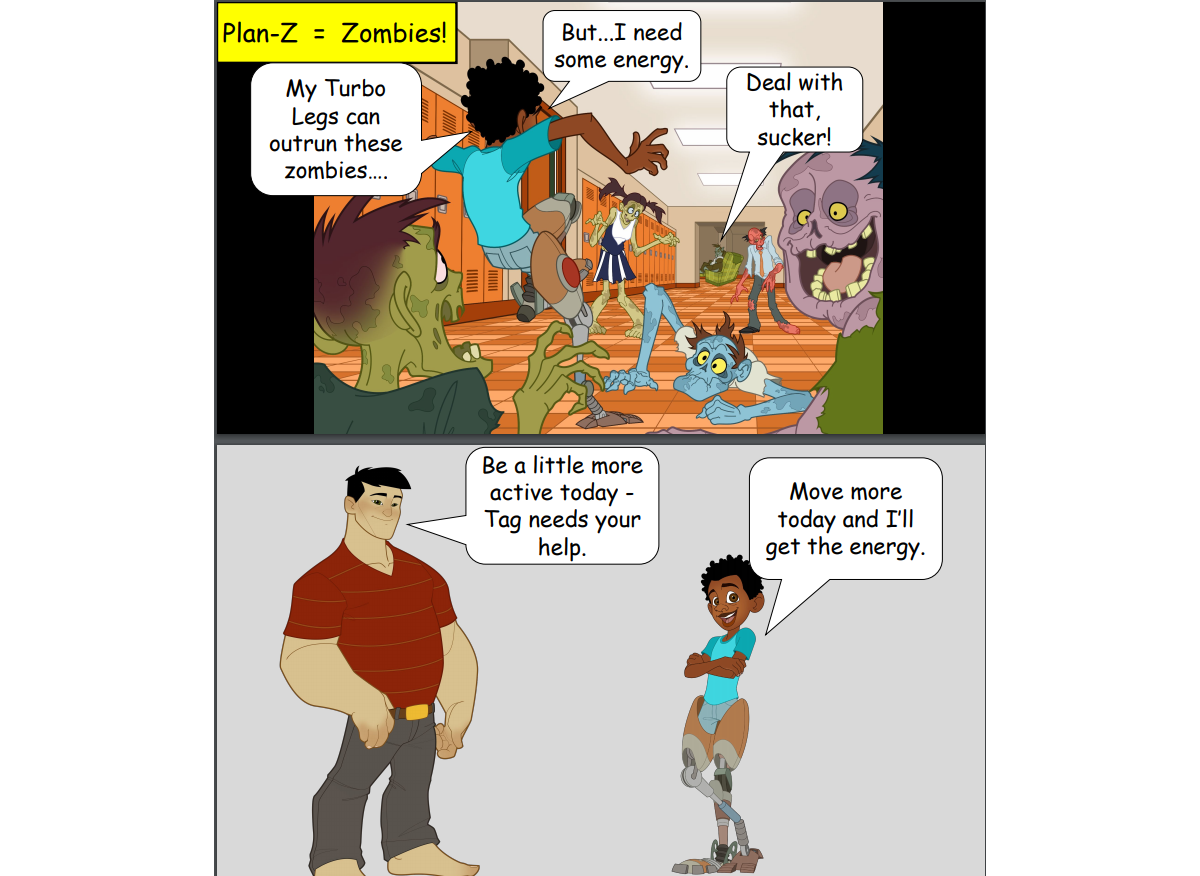
Two sample slides from a FIT Game episode. In the slide prior to the top panel, the villain (shown in the background saying, “Deal with that sucker!”) summoned a group of zombies to prevent the mechanical-legged hero from catching the villain. While the hero is initially confident that his “Turbo Legs can outrun these zombies,” a subsequently revealed voice bubble indicates that he needs energy. The next slide (lower) encourages the children to be a little more active, so that the hero can obtain the energy he needs.

The comic book–formatted FIT Game narrative was presented in slideshow episodes, with a different episode presented each day when PA goals were met the previous day; episodes were displayed on a screen approximately 15 minutes before recess. The top panel of Figure 1 shows one of the narrative slides, and the bottom panel shows a slide in which two of the FITs encourage the sixth-grade participants to help the FITs by being a little more active.

When the intervention began (see below for Experimental Design), children were informed that the object of the game was to help the FITs capture the three members of the VAT. Children were also informed that by collectively meeting or exceeding a daily PA goal, they could assist the FITs by providing them with FIT energy. FIT energy, the children were informed, enabled the FITs to power their ship and other equipment within the narrative of the game. When PA goals were met on day X, the next slideshow episode was shown on day X+1. Thus, the intervention was incentive based such that when the incentives were earned, they were realized within the FIT Game episodes (eg, capturing one of the villains or obtaining a needed piece of equipment).

During the intervention, PA goals were met if the median-per-student step count was at or above the 60th percentile of the preceding 10 days of step counts; for example, on day 11, the goal was the 60th percentile of the median step counts on days 1-10, whereas on day 12, the goal was the 60th percentile of step counts on days 2-11. This percentile schedule of reinforcement [21] uses a moving window of the prior 10 days’ performance to adjust the difficulty of the goal to the current ability of the player (ie, the class). When the PA goals are met, the percentile schedule gradually increases the goal. This maneuver prevents the game from becoming too easy while maintaining a constant difficulty level (at the 60th percentile) throughout the intervention. Likewise, if the children fail to meet their goal, the percentile schedule decreases the next PA goal to reduce the probability that players will become frustrated with persistent failures. On days when the PA goal was not met on the previous day, a new episode was not presented; instead, the teacher informed the students that the next episode would not be presented until they met their goal (which, unbeknownst to them, was adjusted by the percentile schedule of reinforcement). Over the course of the intervention phases (see below for Experimental Design), the class helped the FITs foil the plans of the VAT, find and capture the villains, and save their school in a “boss battle” against the leader of the VAT.

### Experimental Design

A single-case experimental design was employed; specifically, the ABAB reversal design, with “A” referring to pre/postintervention baseline phases and “B,” to the FIT Game phases. This experimental design establishes internal validity by repeatedly manipulating the independent variable and documenting that behavior changes systematically between phases [22-24]. Throughout the experiment, the classroom teacher instructed children to put on their accelerometer in the morning upon arrival (8:30 am) and take it off just before leaving school (2:30 pm); thus, PA levels reported are restricted to activity during school hours. During evening hours, study personnel collected data from each accelerometer and charged it as necessary. Prior to the first baseline phase, students wore the accelerometer for 5 days. This was designed to acclimate children to the device and to reduce the probability of reactivity.

#### Baseline 1 (Days 1-10, 2 School Weeks)

During the Baseline 1 phase, children wore their assigned accelerometers, as described above. The teacher provided no encouragement for children to be more active nor were the children informed of their individual step counts.

#### FIT Game Phase 1 (Days 11-20)

During this phase, procedures continued as outlined in Baseline 1, except that the FIT Game intervention was implemented with daily PA goals and game episodes, when earned (see Intervention section above).

#### Baseline 2 (Days 21-30)

On day 21, children were informed that the game would pause for 2 weeks, as the FITs traveled through a wormhole in space, temporarily blocking communication. During Baseline 2, data collection continued in the manner described in Baseline 1.

#### FIT Game Phase 2 (Days 31-40)

On day 31, the game resumed where it left off, with a new episode presented to the class. As in the prior FIT Game phase, PA was measured daily and new episodes were contingent upon meeting PA goals set in the manner described above.

### Statistical Analysis

Shapiro-Wilk tests indicated that distributions of step counts deviated from normality at the baseline (*W*=0.92; *P=*.03) and during the intervention (*W*=0.93; *P=*.056). Therefore, the Friedman test (nonparametric equivalent of a repeated-measures one-way analysis of variance) was used to determine if differences existed between phases. Comparisons between phases were made using Bonferroni corrected posthoc Wilcoxon matched-pairs signed rank tests (α=0.0083). Effect size was calculated as *r=* *Z/* √N, with values of 0.1, 0.3, and 0.5 commonly interpreted as small, medium, and large effect sizes, respectively [25]. All analyses were conducted using Prism 8.0 for Mac (GraphPad Software Inc, San Diego, CA).

## Results

**Figure 2:**
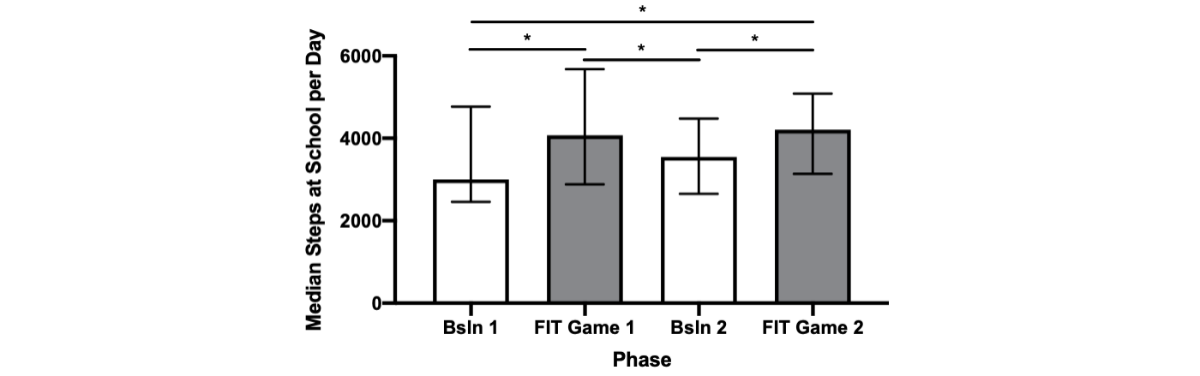
Median (interquartile range) number of steps taken during school hours during baseline and while playing the FIT Game. Bonferroni post-hoc Wilcoxon matched-pairs signed rank test. *P<.001. Bsln: baseline.

Figure 2 shows the median (and interquartile range) steps taken per child per day during the baseline and FIT Game phases. 

Children met their PA goals on 16 of the 20 days (80%) of the FIT Game phases, increasing their median step counts from 3331 per day during baseline to 4102 per day during the FIT Game phases (*X*^2^=39.0; *P<*.001) *.* From Baseline 1 to FIT Game phase 1, PA increased significantly by a median of 1073 steps per child per day (*W*=425; *P*<.001; *r=* 0.603). During Baseline 2, the average number of steps significantly decreased from levels observed in FIT Game phase 1 (*W*=399; *P*<.001; *r=* 0.566), thereby demonstrating experimental control over PA. When the game resumed in FIT Game phase 2, the PA increased significantly above Baseline 2 levels by a median of 658 steps (*W=343*; *P<*.001; *r=* 0.487). Step counts during FIT Game Phase 2 were also significantly higher than Baseline 1 by a median of 1204 steps (*W*=335; *P=*.001; *r=* 0.476) *.* When individual students’ step counts were summed across their two baseline phases and then across their two FIT Game phases, all 29 students increased their PA levels while playing the FIT Game (range: 128-3476 steps; binomial test *P*<.001).

## Discussion

This pilot study illustrated that the FIT Game could be successfully adapted to increase PA at school instead of healthy eating at school. The FIT Game significantly increased PA in sixth-grade students by a median of 771 steps per day at school during the FIT Game phases of the intervention. While the clinical utility of this increase is modest, it should be evaluated in the context of limited opportunities for improvement: Children wore their accelerometers only at school and had only 20 minutes of recess per day. Given the large effect size of this study (median effect size across comparisons: *r*=0.533), future research should evaluate if the FIT Game can produce clinically significant increases in PA (eg, 60 minutes of moderate-to-vigorous activity each day) when accelerometers are worn at school and at home and over a longer period of time. Such an intervention may prove useful in slowing or reversing the reduction in PA that occurs from ages of 6-19 years [13].

Reviews of school-based PA interventions provide a mixed picture of efficacy, with modest effects overall and an overreliance on parent/child self-reports of activity at home [26-28]. Most interventions are designed using Social Cognitive Theory, and some have called for new, goal-based theoretical approaches for developing effective, low-cost, scalable interventions [27]. The FIT Game was developed using operant learning theory and dynamic goal-setting, in which the reinforcement contingency continuously adapts to the “skill” of the player [21]. Reinforcement-based interventions have increased healthy eating in elementary school–aged children, both during the intervention phase and at follow-ups of up to 1 year [29,30]. Therefore, evaluating the longer-lasting effects of the FIT Game on PA outcomes is an important area for future research.

Three limitations of this study are noteworthy. First, from a clinical perspective, it is discouraging that the PA returned to baseline levels when the game was suspended during Baseline 2. We anticipated such decreases, and they were needed to demonstrate the efficacy of the FIT Game within the logic of the single-case experimental design [23]. To address this limitation, future research should employ a cluster randomized design and evaluate the ability of a longer-duration version of the FIT Game to produce behavior change during not only the intervention, but also the postintervention follow-up. Some evidence suggests that health behavior change takes approximately 10 weeks to become habitual [16], suggesting a minimum target for intervention duration. Within the healthy eating literature, long-term interventions using goals and incentives have produced encouraging outcomes [31]. In this pilot study, the FIT Game was played for only 2 weeks before reverting to baseline conditions; therefore, the reversions to baseline PA levels are not surprising.

Second, the study is limited in sample size (N=29) and demographic diversity of the sample (predominantly Caucasian sixth graders living in Northern Utah). Future studies should evaluate the efficacy of the FIT Game on PA in multiple classrooms with more diversity than is represented in this study. Such a study should also attempt to address the third limitation of the present study: PA was measured only during school hours. Practical limitations necessitated this arrangement, as the Fitbit software would not reliably sync all 29 Fitbits during the school day. Solving this measurement issue could positively influence physical activity at home and school, and this is likely to have a larger impact on individual health.

In summary, this project provided initial evidence that the low-cost and low-labor FIT Game could be used to significantly increase elementary school children’s daily levels of PA. Future research should explore if larger increases in PA can be encouraged and maintained over longer intervention phases, as this may produce habitual patterns of activity that could carry on through adulthood. Future research should also explore combining the two versions of the FIT Game, developing one game that simultaneously increases healthy eating and PA each day. Such a low-cost intervention could significantly impact public health if it were widely adopted in developed nations facing a childhood obesity epidemic.

## Abbreviations

FIT: Field Intensive Trainee
PA: physical activity
VAT: Vegetation Annihilation Team
